# A multi-agent large language model framework for intelligent vendor evaluation and risk-aware procurement decisions

**DOI:** 10.1038/s41598-026-50952-x

**Published:** 2026-04-28

**Authors:** Amey Joshi, Mangal Singh, Deepak Parashar, Mahesh Singh

**Affiliations:** 1 Symbiosis Institute of Technology, Pune Campus, Symbiosis International (Deemed University), Pune, India; 2 Department of E&TC, Symbiosis Institute of Technology, Pune Campus, Symbiosis International (Deemed University), Pune, India; 3 Manipal Institute of Technology, Manipal Academy of Higher Education, Manipal, India; 4 Department of Robotics and Automation, Symbiosis Institute of Technology, Pune Campus, Symbiosis International, (Deemed University), Pune, India

**Keywords:** Multi-agent systems, Large language models (LLMs), Vendor selection, Sentiment analysis, Risk assessment, Industry benchmarking, Business and management, Business and management, Information systems and information technology, Mathematics and computing

## Abstract

The process of vendor selection is a multi-dimensional and intricate task that requires analyzing the financial health, amount of risk, industry standards, and the mood in the marketplace. The use of traditional methods that consider individual data points or fixed metrics usually leads to less than optimal procurement decision-making. The current paper introduces a new Multi-Agent Large Language Model (LLM) framework that will improve the process of vendor evaluation based on the analysis of both structured and unstructured data. Its framework uses specialized agents that target different aspects of vendor evaluation: financial analysis, risk profiling, sentiment monitoring, and industry benchmarking. Structured indicators that financial agents consider include liquidity, profitability and solvency. Exposure to geopolitical, operational, and compliance risks is assessed by risk agents. Sentiment agents identify sentiment based on the news articles, reviews, and social media to determine the perception of the people and stakeholders. Agents in benchmarking compare vendors to industry standards to determine outliers and best-in-class vendors. The combination of these insights provides a data-driven yet context-aware procurement recommendation generated by the framework. The qualitative assessments are used to complement quantitative metrics, allowing procurement teams to make informed and holistic decisions. The system does not only help in identifying the most appropriate vendors but also assists in managing relationships with suppliers in the long term whereby the system alerts of any risk and performance deviations in real time. The method enables organizations to concentrate vendor selection in tandem with strategic objectives, reduce risk, and enhance the long-term value. The suggested multi-Agent LLM scheme is a major development in smart procurement systems, which enhance more resilient and value-oriented supply chains.

## Introduction

**Fig. 1:**
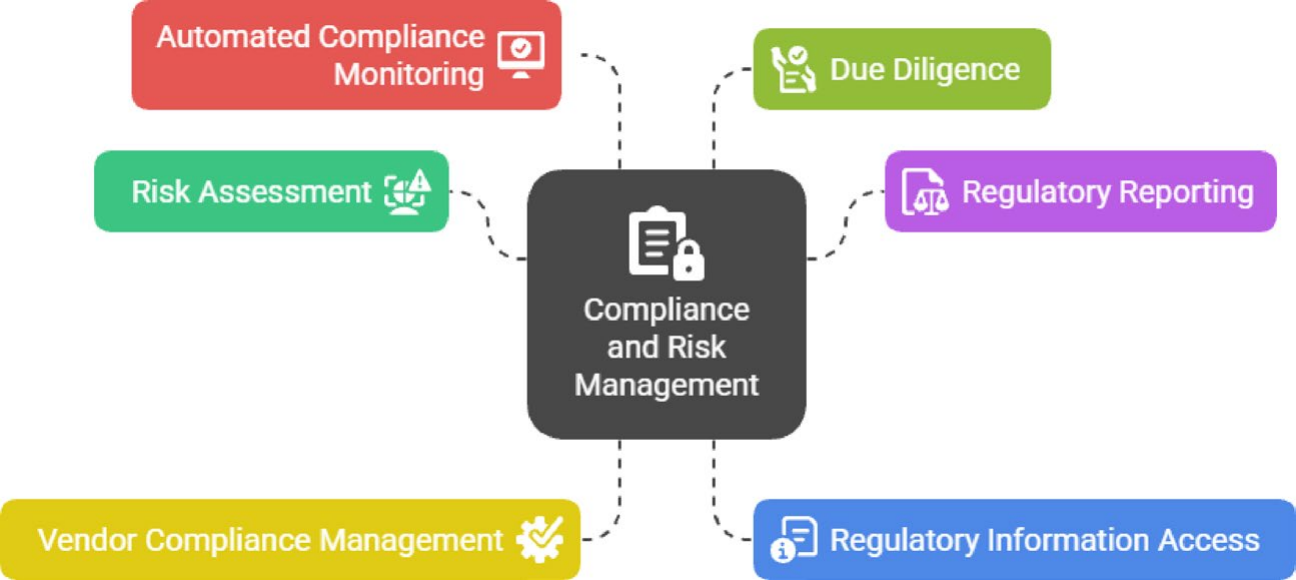
Key components of a vendor compliance and risk management framework.

One of the major components of procurement and supply chain management is the vendor selection, since it assists an organization in becoming more efficient, cost-managed and risk-addressed. You also most frequently check the finances of vendors, their past performance and compliance with standards, but these aspects are not always updated quickly, so your attention should also be paid to the behavior of the latter in emotional terms or their responses to various risks. The frequent interruptions in the supply chain, the leaders should know the correct data and consider various angles to choose the most suitable vendors^1^. Figure 1 shows six essential elements of a successful vendor compliance and procurement management system. It starts with Risk Assessment, during which possible financial, operational, or reputational risks posed by vendors are pointed out and assessed. Regulatory Reporting will ensure that all the necessary documentation and compliance reports are prepared correctly to both internal and external stakeholders. Vendor Compliance Management is a process that includes continuous monitoring of suppliers to make sure that they meet legal and organizational standards. Regulatory Information Access helps the procurement teams have access to current regulatory guidelines and alerts. Automated Compliance Monitoring uses technology to monitor the behavior of vendors constantly and alert to anomalies or risk in real time. Lastly, Due Diligence is considered an extensive pre-engagement due diligence such as audits, background checks, and financial audits. All these components make up a complete supplier risk management system, compliance, and better procurement decisions.

The Procurement and sourcing solutions powered by Artificial Intelligence (AI) have provided a different and innovative way of evaluating and selecting vendors, with sophisticated data analytics and machine learning (ML) algorithms. Although the Large Language Models (LLM) models (such as GPT and BERT) have enhanced financial analysis capabilities in text format, but are still immature in terms of domain-specific vendor profiling, adaptive risk assessment, and multi-source data integration^2^. Conventional LLM methods have problems with the fragmented processing of data and thus they are reactive and not predictive in the process of making procurement decisions.

In order to overcome the limitations discussed above, this paper proposes a Multi-Agent LLM Framework of vendor selection and strategic sourcing, which is a combination of six specialized agents assessing and evaluating various criteria of vendor evaluation. Agents are concerned with the financial health of the supplier, risk evaluation, market sentiments analysis, benchmarking on the industry, optimization of sourcing and optimization of procurement strategies. Through such a multi-agent architecture, organizations are now able to handle extensive structured and unstructured procurement data, vendor selection decisions are scalable, risk-conscious and strategic-oriented.

**Fig. 2:**
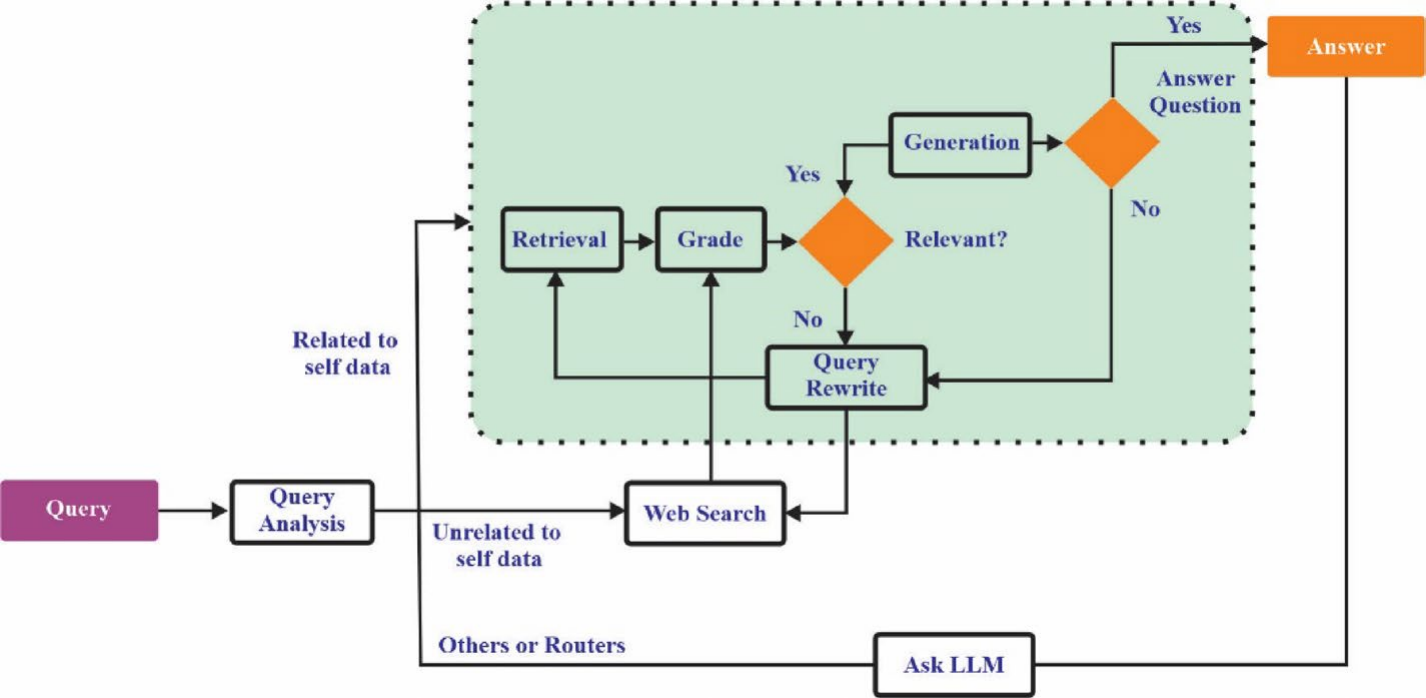
Workflow of a question-answering system, from query input to final answer generation.

This flowchart of Fig. 2 shows the decision-making process in a question-answering system. It begins with a user query, which undergoes query analysis to determine if it’s related to self-data^3^. If it is, the system retrieves relevant data, grades its usefulness, and checks if it’s relevant. If so, the system generates an answer and checks if it adequately answers the question; if yes, it returns the answer. If not relevant, the query is rewritten and the process loops. If the query is unrelated to self data, a web search is conducted or the query is routed to a large language model (LLM) for a response.

This multi-agent LLM framework enhances vendor selection and sourcing intelligence by incorporating quantitative and qualitative evaluation metrics (e.g., BLEU, METEOR, BERT Score for linguistic and analytical accuracy) and qualitative assessments (e.g., coherence, relevance, risk integration, sentiment alignment) to provide comprehensive, interpretable vendor recommendations^4^. By leveraging multi-agent reasoning, organizations can optimize supplier relationships, mitigate procurement risks, and improve long-term sourcing efficiency.

The combination of MAS and LLMs is now improving the efficiency of making decisions, carefully assessing risks and automating negotiations in managing the supply chain^5^. The usual process of procurement relies on strong financial benchmarks and manual review on supplier’s performance, however, this leaves a little room for flexibility during changes in markets. Alternatively, the Multi-Agent System framework improves the way suppliers are chosen by making agents collaborate without direction, analyzing recent data automatically and perfecting negotiated contracts with AI. Currently, researchers are placing more focus on MARL, using deep learning to evaluate suppliers and rate companies according to their sentiment. This section concentrates on main publications on multi-agent decision systems related to sourcing, AI and supplier negotiations, as well as the risks incorporated.

Choosing the right supplier is one of the most critical tasks in procurement, requiring multi-dimensional assessments, including financial health, compliance ratings, risk exposure, and competitive positioning.^6^ introduced a new way to use AI so that MAS is capable of reviewing vendor data and performing risk modeling on its own to evaluate potential vendors. Adaptive algorithms are integrated into their system to measure how trustworthy certain suppliers have been when compared to previous purchasing behavior. Liu et al.^7^ improved this concept by designing a multi-agent collaborative framework for supplier selection in unclear circumstances, incorporating real-time market fluctuations and operational risk modeling. Their research validates how MAS dynamically adjusts sourcing decisions, ensuring procurement choices align with strategic financial benchmarks. Kim and Choi^8^ developed an automated negotiation system that allows vendors to improve their flexibility when contracting with suppliers. Research shows that their method of negotiations yields superior agreements with suppliers, fewer ineffective parts of contracts and more confident decisions. In their research,^9^ looked into multi-agent reinforcement learning (MARL) when handling negotiations among self-governing suppliers. They also show that AI-assisted MAS systems can refine their sourcing processes using instant data received during procurement. The study on MAS explores improvements in handling contracting and cutting costs, while lowering the purchase-related workload and improving relationships with suppliers. With strategic sourcing, you prepare for future purchases, handle your payment budget, monitor the quality of your vendors and try to minimize risks^10^. investigated using AI for strategic sourcing by creating models that allow AI to review vendor information. They highlight that AI use by companies helps safeguard their buying and reduces risks in the supply chain^11^ introduced agents to observe which risks are confronting vendors, through the use of deep learning to find incidents that involve cash problems, rule breaches and irregular conduct. Experts have found that the use of AI in MAS helps a company reduce risks in their procurement since suppliers comply more and fewer issues occur. Wang and Lin^12^ explored the process of choosing the right suppliers in AI-enabled procurement. This emphasizes using machine intelligence to reduce the dangers of relying on unverified suppliers. It confirms that MAS is necessary for gathering procurement intelligence, allowing vendors’ evaluation systems to adjust to new developments in the market^13^. created models for AI-powered sourcing that support the supply chain by enabling smarter vendor selection in the face of economic changes. What they found is that providing teams with flexible capabilities helps them fine-tune their strategies for managing expenses. MAS involves studying business attitudes in procurement and uses that insight to pick safer suppliers within the market. In Park and Lee^14^ used an AI system with multiple agents to assess supplier performance by analyzing sentiment to check on a vendor’s reliability and credibility. The authors of this study^15^ introduced a model that uses both real-time sentiment in media and financial information to help avoid working with high-risk suppliers. The model demonstrates that using sentiment intelligence can improve the way a company picks suppliers and meets the standards indicated by industry and investors.

As a result of these studies, it is now evident that knowing how vendors feel is valuable since MAS can quickly judge the reliability of suppliers. Combining deep learning models, autonomous assessments and supplier selection focused on the market makes it crucial to use collaborative MAS frameworks for improving supply chain intelligence^16^. write that AI improves the process of benchmarking supplies by using the results from multi-agent work in the financial sector. Li and his team proposed deep learning for multi-agent supplier evaluation and found that it improves the decision alignment of agents as well as boosts the accuracy of identifying suppliers^17^. Researchers found that combining MAS and deep learning analytics supports algorithmic intelligence in the supplier selection process^18^] explored multi-agent procurement agility, proposing MAS-driven adaptability mechanisms for optimizing sourcing strategies in volatile markets. Their findings suggest MAS improves supplier diversity selection, enhances procurement stability, and refines risk-aware procurement intelligence.

Existing research on multi-agent systems in procurement intelligence highlights their role in automating supplier selection, refining sourcing decisions, and enhancing negotiation efficiency. By leveraging reinforcement learning, sentiment analysis, and AI-driven financial models, MAS-driven frameworks significantly reduce procurement risks while improving sourcing agility. Future studies should explore advanced hybrid MAS models integrating deep learning, further optimizing strategic procurement intelligence and adaptive sourcing decision-making^19^.

Although there are benefits to individual perspectives, the conventional methods are likely to be fragmented, passive and responsive—this constrains their capacity to combine multiple data streams to make lucrative investments. The conventional approach will tend to overlook cross-industrial connections of financial indicator, which consequently compromises the decision-making process. As an example, the volatility index (VIX), which is also referred to as the fear gauge, is widely applied to risk measurement, yet rarely applied in conjunction with sentiment information that is derived based on news reports, earnings conferences or internet messages. Without the capability of integrating these elements, current technologies decrease the precision and universal range of stock suggestions frameworks.

The degree of advancement of giants of large language models like GPT and BERT derivatives has substantially revolutionized NLP to permit the formation of financial insights out of unstructured raw documents in finance. These models have facilitated the summary of the earnings debate, the detection of sentiment in news articles and the forecasting of the line of direction in stock on the basis of sentiment changes amid investors. Yet, the models also have significant weaknesses when applied in isolation: they tend to be bad at specializing in particular areas of finance when trained on broad datasets not related to any particular industry, they can be susceptible to errors when analyzing temporal trends in dynamic markets, and they are not designed to handle different types of financial data simultaneously^20^.

The suggested system will include six agents which are constrained to work on the data both in parallel and in hierarchical mode. Operating simultaneously, Fundamental, Technical, Risk and Sentiment Analysis Agents, generate particular data-driven information. By adhering to a hierarchical strategy the Industry Sector & Competitor Agent is able to incorporate the information provided by the rest of the agents to give detailed evaluations of the business trends in the industry in addition to that of a competitor. The Strategy Expert Agent is at the peak of the hierarchy, combining all the accrued knowledge to provide straightforward and unwavering advice on investments, as the dust settles at the end of decisions. With this mixed processing technique, the system can achieve the efficiency of modularity besides smooth integration of domains hence making the system highly marketable.

This model relies on an in-depth method of the assessment to ascertain the dependability and impact of stock suggestions. To measure the faithfulness of the knowledge, extracted by the agents against the expert references, this framework uses BLEU, METEOR, BERT Score, and Cosine Similarity to both test the existence of linguistic coherence and the semantic compatibility. Financial decisions are determined with sentiment alignment and risk deviation indicators, which ensure consistency between investment advice and market expectations and risks taken. The recommendations given by agents are evaluated against historical market data as well as their financial forecasts.

The paper introduces a novel Multi- Agent LLM Framework of Stock Recommendation through Multi-Modal Signal Integration and efficient optimization of structured and unstructured financial data processing to guide a better investment choice. The work has made the following contributions: the creation of a specialized multi-agent system to analyze financial data; the improvement of domain-specific stock analyses. Another technology that is presented through the framework is agent-based collaborative reasoning, which guarantees interpretability and dynamism in real-time investment advice. Lastly, the system is tested on sound evaluation metrics, which guarantee semantic soundness, financial integrity, and sentiment-driven suggestions to assist strategic investment choices.

The rest of this paper is structured in the following way. "Proposed multi-agent framework" section outlines the suggested multi-agent system, agent roles, and specialization, agent interaction, and training procedures. "Data sources & preprocessing techniques" section describes data sources and pre-processing methods. "System evaluation & benchmarking" section talks about system evaluations with qualitative and quantitative methods, empirical benchmarks and analysis of investment in the study. Lastly, "Conclusion" section concludes with insights, limitations and future directions of developing multi-agent financial decision-support systems.

## Proposed multi-agent framework

**Fig. 3:**
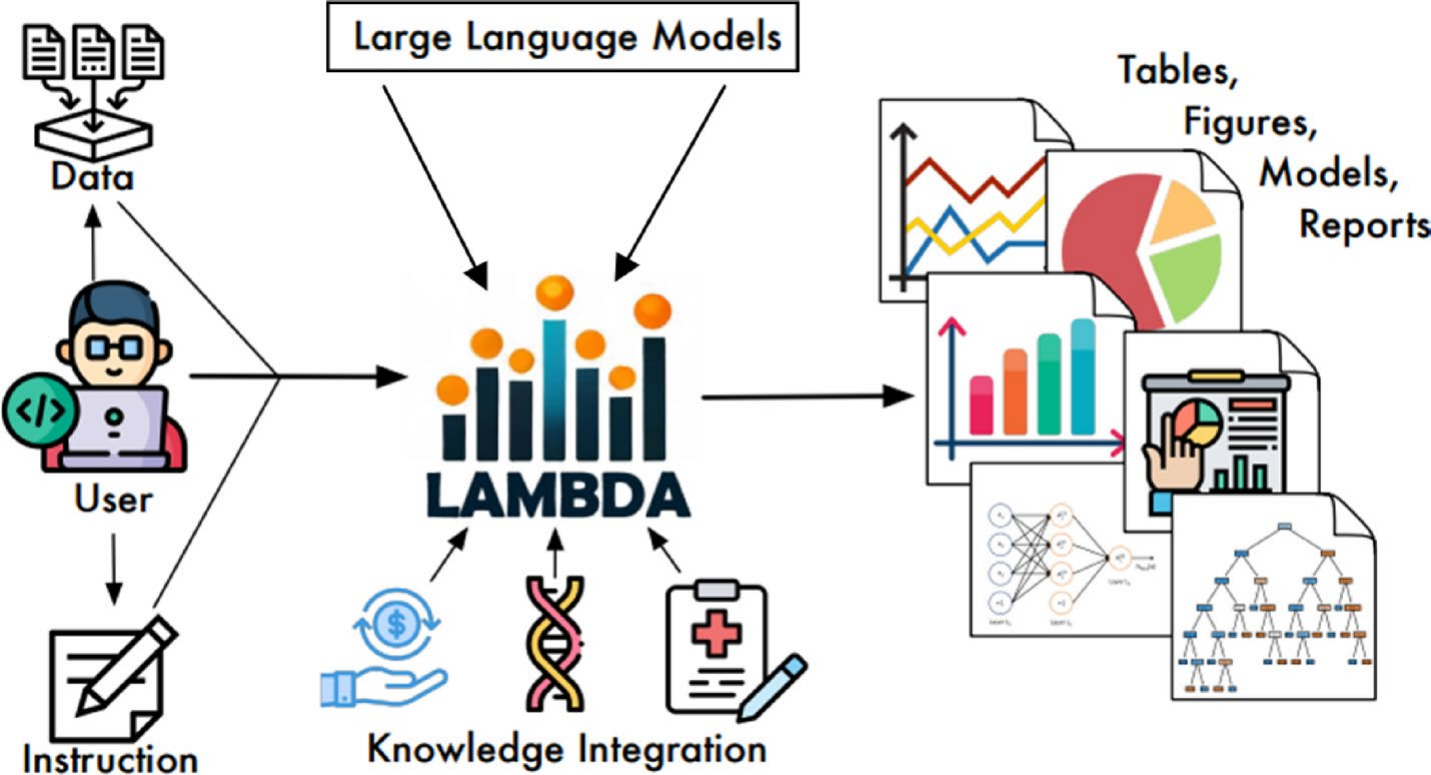
Overview of the LAMBDA framework using generic Large Language Models (LLMs) to generate structured outputs.

With the Multi-Agent Large Language Model (LLM), procurement is likely to be more efficient as it involves multiple agents that look at distinct sides of vendor analysis. Instead of relying on financial ratios or their subjective assessment, this time we make decisions based on data to determine financial stability, identify risks, see trends, compare with industries, look at sourcing and select the most appropriate vendors. Architecture of the proposed LAMBDA framework integrating data, user inputs, instructions, knowledge sources, and Large Language Models (LLMs) to generate analytical outputs in depicted in Fig. 3. In its simplest form, LAMBDA is an integration of the capabilities of these models and the domain-specific knowledge of finance, healthcare, and science to provide structured, insight-rich outputs^21^. The system is used by providing data and prompts, which are processed by LAMBDA using a multi-step pipeline that incorporates natural language understanding, domain knowledge integration, and reasoning. The result is an assortment of practical products such as tables, charts, decision models and analytical reports. This framework shows how LLMs can be implemented as a decision-support tool that can transform complex data into practical knowledge in domains.

All the vendors are evaluated individually, as there is a system where all the evaluations combine and justify the purchasing objectives. Combining structured data analysis with unstructured data on opinion, this architecture helps to choose the appropriate suppliers, minimizing risks and maximizing efficiency in sourcing.

**Fig. 4:**
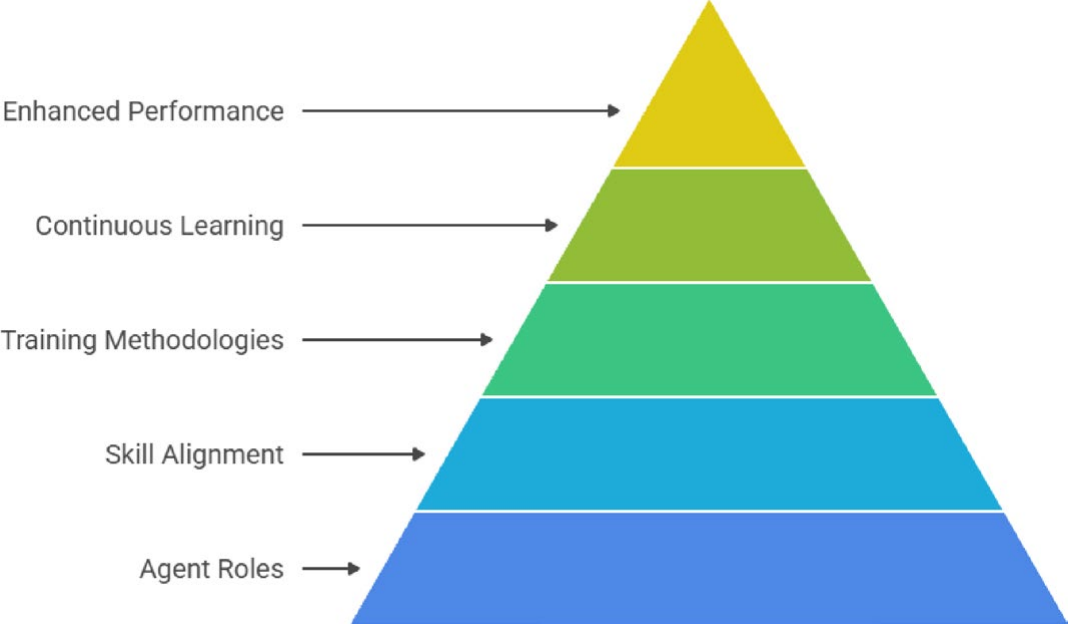
A hierarchical model illustrating the foundational layers.

The Fig. 4 demonstrates the stratified background that is required to realize improved functionality in a multi-agent or AI-based system. At the bottom, there are well-defined Agent Roles that guarantee specialization of tasks and functional distinctiveness. Expanding on that, Skill Alignment is the process that pairs the agents with the skills necessary to be successful in their work.

The next level is Training Methodologies which are concerned with the mode of learning by agents using supervised methods, reinforcement learning methods, or prompt tuning. This results in Continuous Learning in which agents evolve through time as they incorporate feedback and new information^22^. The final product of a properly designed system, which is constantly changing, is at the apex, Enhanced Performance. The model highlights that agent systems that are intelligent and perform well require a robust initial design.

### Agent roles & specializations

The framework works by bringing together five agents that are responsible for separate parts of checking financial risk, tracking sentiment and choosing vendors. Together, these agents improve procurement intelligence, handle risks from vendors and give strong advice on which suppliers to work with.: Does the Company Have the Money to Succeed?

1. The Financial Viability Agent evaluates a vendor’s finances, liquidity and capacity for sustainability to guarantee steady relationships with suppliers in the future. Going through detailed financial records, it gently assists procurement managers in choosing the best supplier for their needs. Indicators Looking at Finances Considered.
  Liquidity Measures: Debt-to-Equity Ratio, Current Ratio and Quick Ratio used to evaluate how stable a company’s finances are. Likewise, you need to analyze Profitability by checking Return on Assets (ROA), Gross Margin and Operating Margin to evaluate supplier efficiency.Understanding the cash flow: Free Cash Flow (FCF) and monitoring changes in Net Income to assess chances and risks of reinvestment.

2. Risk Exposure Agent, exposure Agent studies the financial and working risks linked to vendors, making sure procurement efforts are in line with reducing risks. Market stability, following regulations and reactions to external supply chain issues are examined to ensure purchases remain reliable. The Risk Metrics Looked at Are: Monitoring risk changes in markets: using Beta, Value-at-Risk (VaR) and Maximum Drawdown.
  It’s important to oversee suppliers regarding regulatory obligations, authorizations and ratings because they influence whether the industry process follows guidelines. Risks related to the supply chain such as disruptions or global political issues, all need to be monitored.

3. Sentiment Analysis Agent: The agent analyzes real-time responses to collect market opinions, sector feedback and insights into investors’ attitudes, using them to accurately reflect how well vendors are trusted. Some of the Top Sentiment Metrics Under Review:Analyzing the Reputation of Vendors: We use Google News RSS feeds, reviews by analysts and study changes in public perception and media coverage. Machine learning tools analyze the discussions, latest financial news and popular trends linked to an industry. Sentiment scoring reviews a vendor’s stability and estimates the market’s response to the vendor.Using Sentiment Analysis, the agent keeps an eye on current market opinions and opinions from the industry to include vendor reputation in the procurement recommendations^23^.

Types of Sentiment Metrics Analysis Included:

- Looking at how vendors are perceived: Analysis of Google News for current news, analyst reviews and an assessment of sentiment trending.
- AI technology follows conversations, industry news and key trends across the sector.
- Vendor Reliability & Sector Response: Sentiment scoring rates a vendor’s security and shows how stakeholders might react to events.

The sentiment score moves from negative (−1) to positive (+1) and Valence represents the set of predefined emotions given to words. The intensity selector works by adjusting the valence depending on negation, how much something is emphasized and where capital letters are used.

Alpha helps keep results stable by cutting down on biases and correctly weighing all sentiments using Eq. (1)

1 $${\text{Sentiment Score}} = \frac{{\sum \left( {valence \times intensity} \right)}}{{\sqrt {\sum \left( {valence^{2} } \right) + \alpha } }}$$

4. Industry Benchmarking Agent: The Industry Benchmarking Agent checks vendor prices, services and operational efficiency against other companies to ensure the organization buys at the expected and proper levels.
  The following has been audited and compared:
  Competitor analysis in certain industry sectors is part of market positioning.
  Analyses the cost benefits offered by competing suppliers.
  How effectively has the operation met its promised completion dates, maintained SLA requirements and answered suppliers’ questions?
5. Strategy & Decision Agent: Through combining all agent feedback, the Strategy & Decision Agent develops the best recommendations for sourcing that conform to the company’s aims, budget limits and what’s happening in the market.
6. Decision-Making Process:
  Multi-Agent Data Synthesis integrates finance, risk, sentiment and benchmark data into a clear procurement strategy.
  The report makes practical recommendations for choosing suppliers by assessing many factors.
  Managing procurement strategy well: Gives a company a cost advantage, helps reduce risks and guarantees reliable supplier partners.

### Agent interaction

By adopting both parallel and hierarchical methods, Multi-Agent LLM Framework makes sure its decisions in finance are accurate, efficient and flexible. The system makes it possible for agents to handle their own tasks as they support each other to produce reliable financial judgments^24^.

#### Parallel processing

The framework supports running tasks in different areas by giving Financial Viability, Risk Exposure, Sentiment Analysis and Industry Benchmarking Agents their own space to operate. Each agent separates important data concerning suppliers, market changes and risk, so it offers real-time updates without relying on other agents.

####  Hierarchical processing

At its basis, the Strategy & Decision Agent combines the output of the four parallel agents into logical and thorough advice on procurement decisions. The agent improves insights by bringing sector benchmarks, a comparison of competitors and risk evaluations to the analysis. Following the process guarantees procurement managers have access to the best and safest investment advice for their companies.

Using this hybrid model, the framework quickly responds to market changes, supplying clear, accurate and data-based financial intelligence for assessing vendors and planning investments.

### Training methodologies

By using Llama 2 at the same location as Yfinance information, predictions can be made more accurately and smoothly^25^. By using Llama 2, each agent reasons mainly and analyzesparticular sectors for right decision-making. Operating models at the local level makes data safer, improves how fast inferences can be made and aids the process of integrating into markets. Because of engineering, the system can modify its assessments according to current monetary conditions, making it unnecessary to run complicated revisions.

## Data sources & preprocessing techniques

**Fig. 5:**
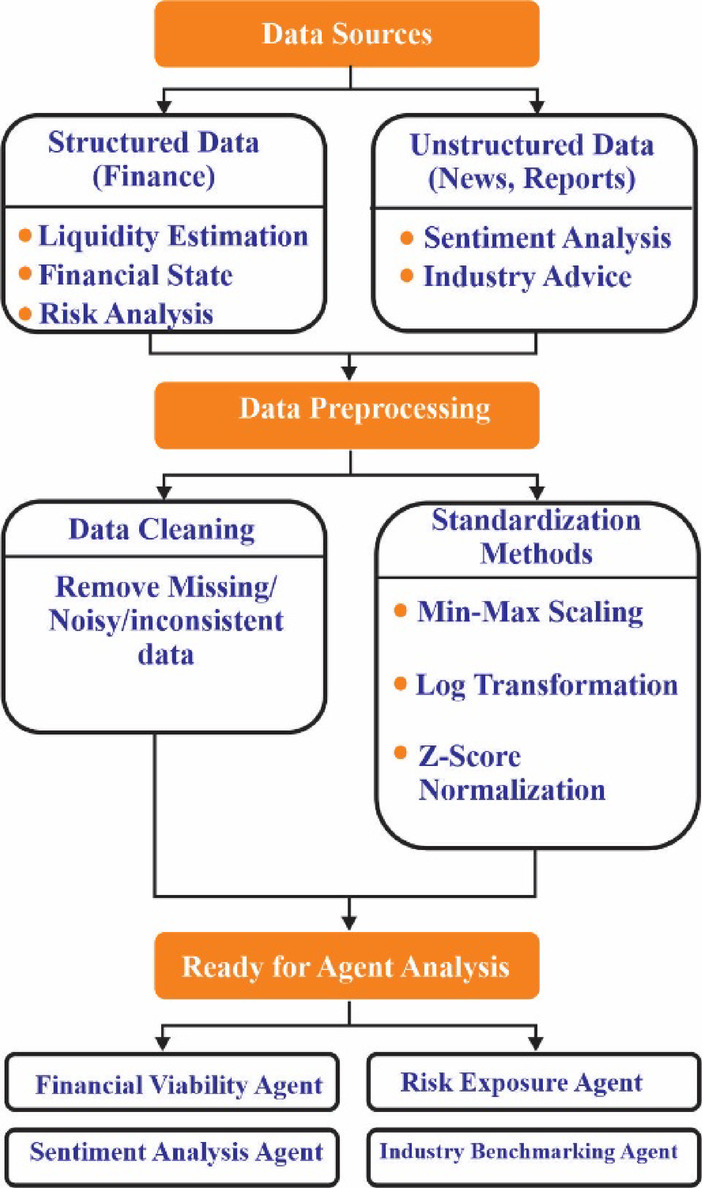
Flow chart of data processing.

What You Need to Know and Steps to Take for Data Processing. The Multi-Agent LLM Framework works well in choosing and buying from vendors when their financial and operating data is up-to-date and correct. The flow chart is shoe in Fig. 5.

It covers the sources for agent data, shows the processes used to maintain data consistency and discusses methods for handling financial data used in analysis.

1. Data Sources: Within both structured and unstructured dataset collections, the framework checks vendors by studying data available from finance^26^. Based on numbers, LFI estimates liquidity, ongoing financial state and what risks may emerge. These assets support the work of the Financial Viability, Risk Exposure, Sentiment Analysis and Industry Benchmarking Agents in their analysis.
  Unstructured data consists of news and analyst reports which help analysts conduct sentiment analysis and learn about industry advice.
  Measuring data and analyzing what is being said, the model allows for a detailed view of vendors that fits today’s procurement practices and competitor actions.

2. Data Preprocessing is the term for several ways to prepare data before analysis.
  Having a framework improves both how correct data is and the accuracy of analytics as it cleans up any missing, noisy or inconsistent information in financial data before analysis.

3. Methods of Improving and Repairing Data.
  It uses the past averages of the data to fill in gaps and preserves the usual pattern in the series. Reviewing data using statistics removes anomalies and leads to a more accurate understanding of what the records show. Building standards and making sure things are the same is the other main challenge.Using Min-Max Scaling, data from financial time series is adjusted so it is easier to compare price changes and higher volatility. Log Transformation corrects distortions in financial ratios and gives useful insight into a firm’s earnings and who its suppliers are.All important risks are made standard through Z-Score Normalization which allows an accurate comparison of all types of spending.

## System evaluation & benchmarking

To evaluate the efficiency and the dependability of the suggested multi-agent LLM framework in terms of vendor selection, the entire system was evaluated and benchmarked. This entailed qualitative and quantitative evaluations to confirm the performance of single agents and the entire structure. To assess the relevance, coherence, and clarity of the generated outputs, the evaluation targeted such important linguistic and analytical metrics as BLEU, METEOR, BERTScore, and readability. Furthermore, vendor-specific benchmarking was conducted through comparison of the outputs of the system with professional curated benchmarks in key dimensions such as financial accuracy, risk profiling, sentiment alignment, and industry benchmarking. This twin-layered assessment system was critical in the sense that the system was not only generating linguistically robust results but it was also providing insights, which were consistent with the expert judgment and procurement goals.

### Assessing the accuracy of choosing the right vendor

The vendor-selection suggestions of the framework are enhanced to be dependable, impartial and risk-free by gauging them using a number of metrics. In order to select the vendors of Ajanta Pharma, Piramal Pharma and Dr. Lal Path Labs, we apply multi-agent AI to study the financial capacity of each company, its risks, the tendencies in the social opinion and position in the market^27^.

Tests are undertaken to ensure the information is co-ordinated, whole, useful, deals with risks and is in the same mood.

Instead of actively concentrating on active trades, JSF and ES analyze specific scores, identify the utilization of similar investment strategies and monitor these changes in the risk profile of a fund. We do not only test what we recommend by past examples but also by how vendors do it, to make sure it will work^28^.

### Qualitative evaluation of vendor selection insights

We examine whether AI vendor recommendations are clear, considerate and aligned with the plans of the team.

Table 1 shows a summary of vendor assessment based on five important qualitative aspects of: coherence, accuracy, completeness, risk integration and sentiment alignment. Of the three vendors, Piramal Pharma is the top-ranked in overall scores, specifically in coherence (4.6), accuracy (4.6), and completeness (4.5), which denotes a high profile, well-documented, and reliable and comprehensive information.

**Table 1:** Vendor evaluation summary

Vendor	Coherence	Accuracy	Completeness	Risk integration	Sentiment alignment
Ajanta Pharma	4.2	4.4	4.1	4.0	4.5
Piramal Pharma	4.6	4.6	4.5	4.4	4.3
Dr. Lal Path Labs	4.0	4.1	4.0	3.7	3.9

Ajanta pharma has a good sentiment alignment (4.5) and accuracy (4.4) with good public perception and data reliability. Conversely, Dr. Lal Path Labs scores lowest in most of the dimensions, particularly the risk integration (3.7) and the sentiment alignment (3.9) indicating a more exposed risk and a weaker sentiment alignment. The overall conclusions of these assessments is that Piramal Pharma is the best balanced and performing vendor, with Ajanta Pharma being a close second.

**Fig. 6:**
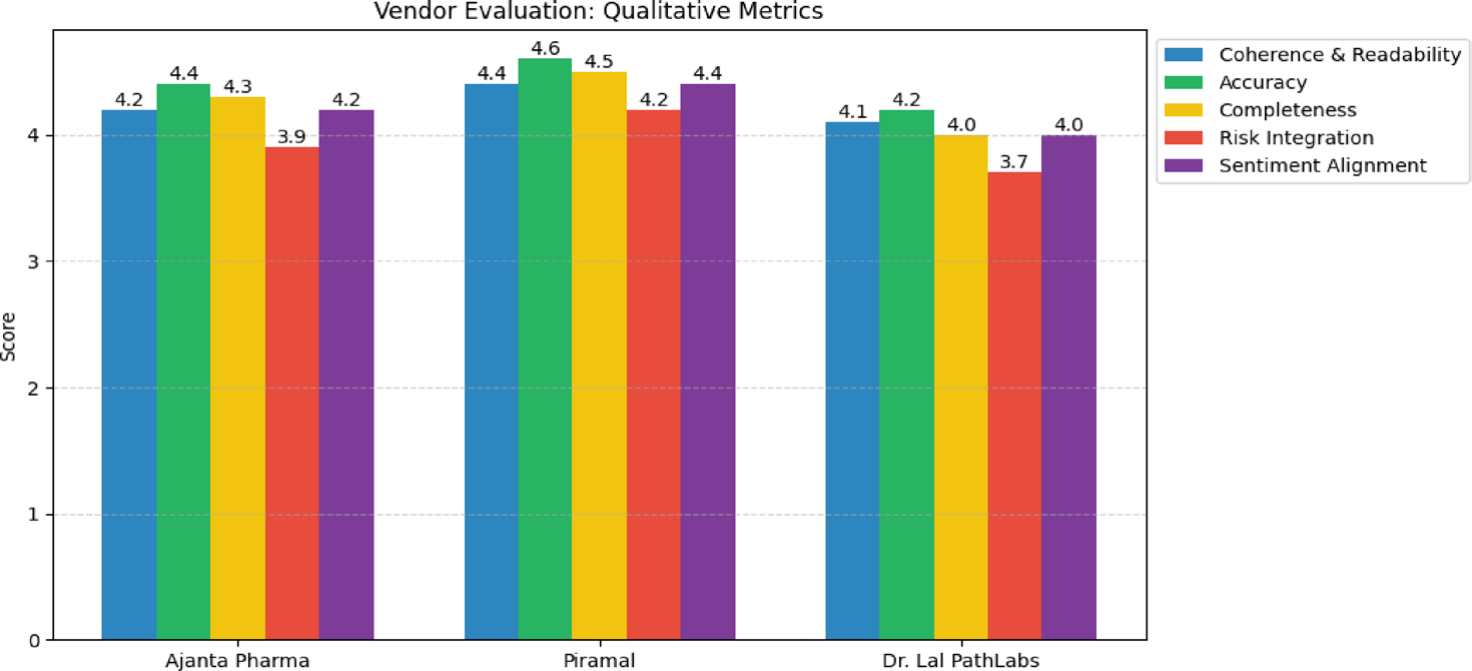
Vendor evaluation based on qualitative metrics.

You can quickly see from the bar chart shown in Fig. 6 which company applies coherence, accuracy, completeness, risk integration and sentiment alignment the best: Ajanta Pharma, Piramal or Dr. Lal Path Labs. All of them demonstrate whether AI-based vendor suggestions agree with procurement intelligence and the market’s consistency. A suitable recommendation for finance management should be clear, logical and finishing with accurate financial details and important procurement reasons. Looking at things like risk allows the company to know how suppliers are handled, whereas looking at sentiment alignment shows if opinions about vendors are following market trends. It turns out that Piramal has the highest and best accuracy (4.6) and completeness (4.5) of all, improving your financial knowledge. At the same time, Ajanta Pharma reflects high levels of investor enthusiasm and good purchasing trustworthiness (4.2).

Dr. Lal PathLabs displays lower risk integration (3.7) indicating that supply chain companies may experience a lot of variation in income. Because we compare options like this, purchase managers get the data they require to judge possible vendors and protect their business interests. In qualitative analysis, we assess if AI vendor guidance is easy to take action on, is clear and fits with the procurement team’s method. You can observe from the bar chart how each company measures against coherence, accuracy, completeness, risk integration and sentiment alignment. All these cases demonstrate how well AI vendor recommendations align with intelligence from the market. The recommendation needs to be simple to understand and structured well and the explanations concerning finance and procurement should match what is stated in the information. Bringing together risks keeps us informed about suppliers and examining sentiment ensures we know if opinions about a vendor’s performance are still in line with what’s happening in the industry.

### Quantitative metrics for vendor recommendation quality

The framework adds numeric analysis to highlight the accuracy of AI in helping choose suppliers, as compared to established procurement requirements.

Table 2 shows the quantitative evaluation metrics by each vendor as per BLEU, METEOR, BERT Score and readability. Piramal Pharma has the highest BERT Score (0.758), meaning that it has a high semantic similarity with reference outputs, whereas Ajanta Pharma has the highest readability (11.42) meaning that the content is clear and more accessible. Despite the highest BLEU (0.19) and METEOR (0.14), Dr. Lal Path Labs is less readable, has a lower BERT Score, which reflects a lower degree of coherence and alignment in general.

**Table 2:** Quantitative metrics.

Vendor	BLEU	METEOR	BERT Score	Readability
Ajanta Pharma	0.17	0.12	0.740	11.42
Piramal Pharma	0.18	0.13	0.758	10.85
Dr. Lal Path Labs	0.19	0.14	0.735	8.79

**Fig. 7:**
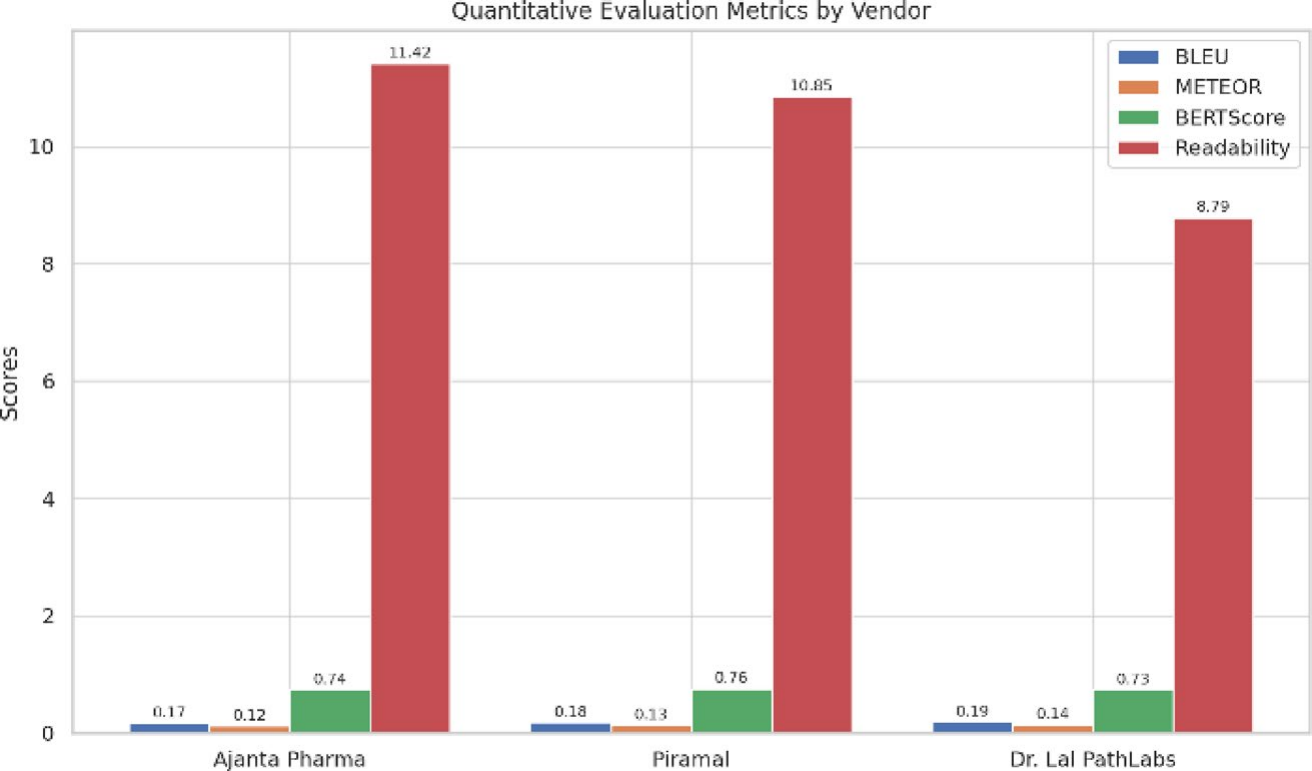
Quantitative evaluation metrics by vendor.

Figure 7 is a bar chart that compares vendors in terms of four quantitative measures: BLEU, METEOR, BERTScore, and Readability. Although all the vendors work equally well on BLEU and METEOR, Piramal demonstrates the best semantic alignment, and Ajanta Pharma is the most successful in terms of clarity in the content. Although Dr. Lal PathLabs scores low in lexical, it lags in readability, implying that its outputs are less readable.

**Fig. 8:**
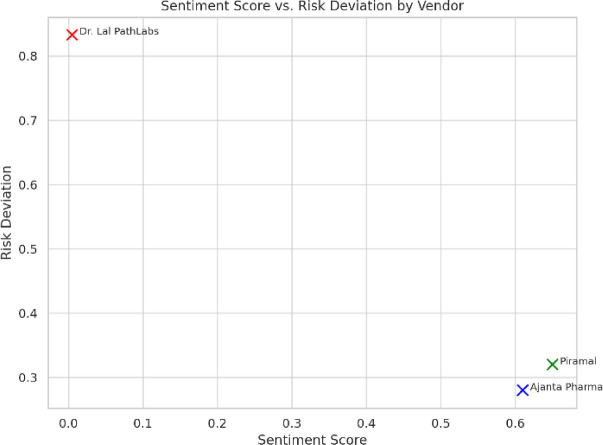
Sentiment Score vs. Risk Deviation by Vendor.

Figure 8 shows the scatter plot of the three vendors in terms of sentiment score and risk deviation. Ajanta Pharma and Piramal depict bigger scores of sentiment (more than 0.6) with smaller risk deviation (approximately 0.28–0.32), which shows positive perception and stability. Conversely, the sentiment score of Dr. Lal Path Labs is very low (almost zero) and risk deviation is much higher (over 0.8), which indicates possible volatility and less confidence of the market. Top scores in BLEU (0.19) and METEOR (0.14) will enable Dr. Lal PathLabs to remain on par with the definer of what is currently being done in the core industry.

The BERT Score assessment ensured that Piramal scored the highest (0.758) which confirmed their scores to be accurate and detailed. Due to its highest readability score (11.42) the insight of Ajanta Pharma is easy to locate and comprehend.

**Fig. 9:**
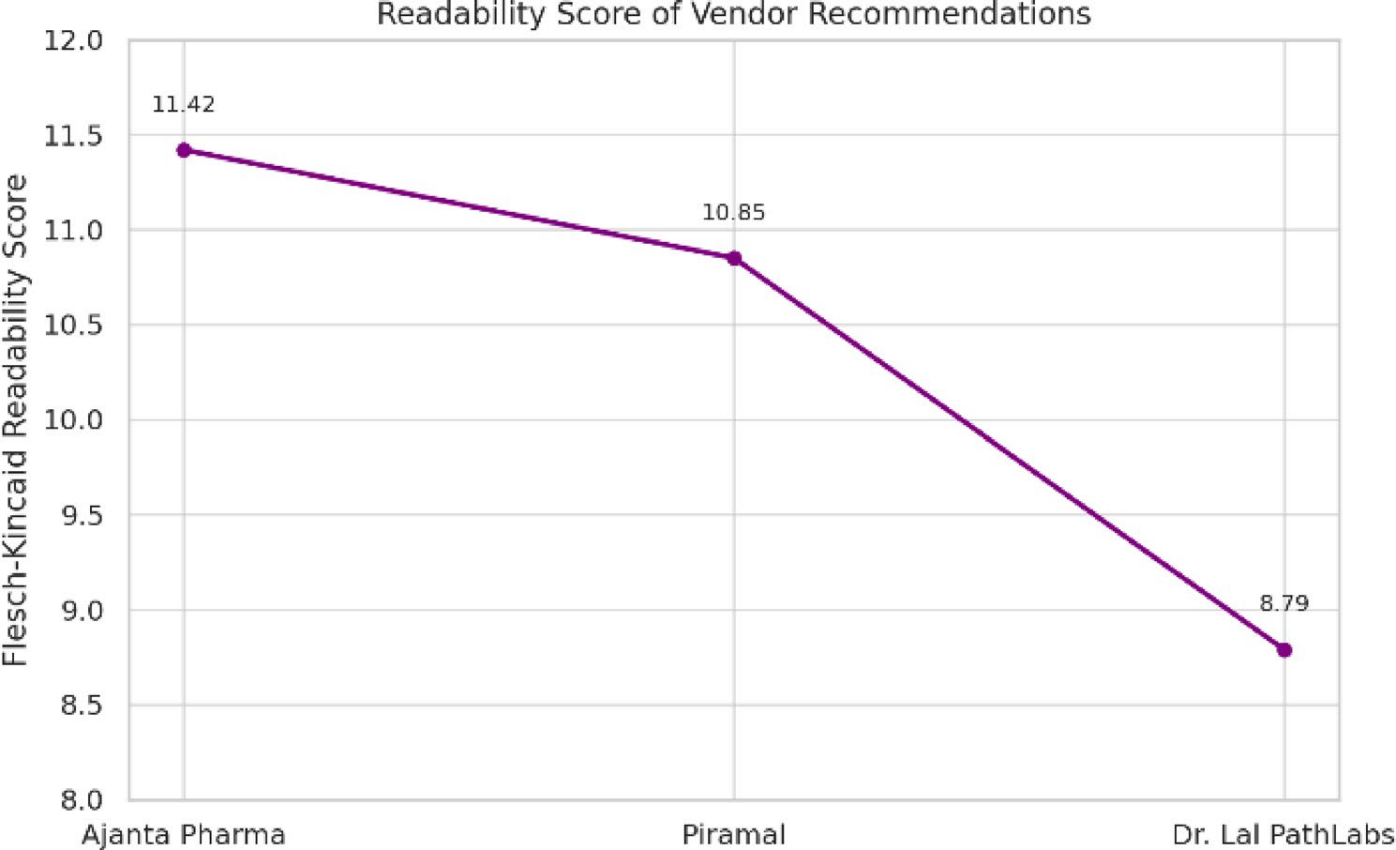
Readability score of vendor recommendations.

In Fig. 9, this line graph verifies the extent of AI-based procurement recommendations usefulness in terms of readability. Ajanta Pharma received the highest rating 11.42 due to offering helpful advice on investors, whereas Dr. Lal PathLabs received a rating of 8.79 indicating that their presentation of supplier ratings are more complex. The scores can be easily located courtesy of the data labels and the graph is kept clean courtesy of the minimal design.

**Fig. 10:**
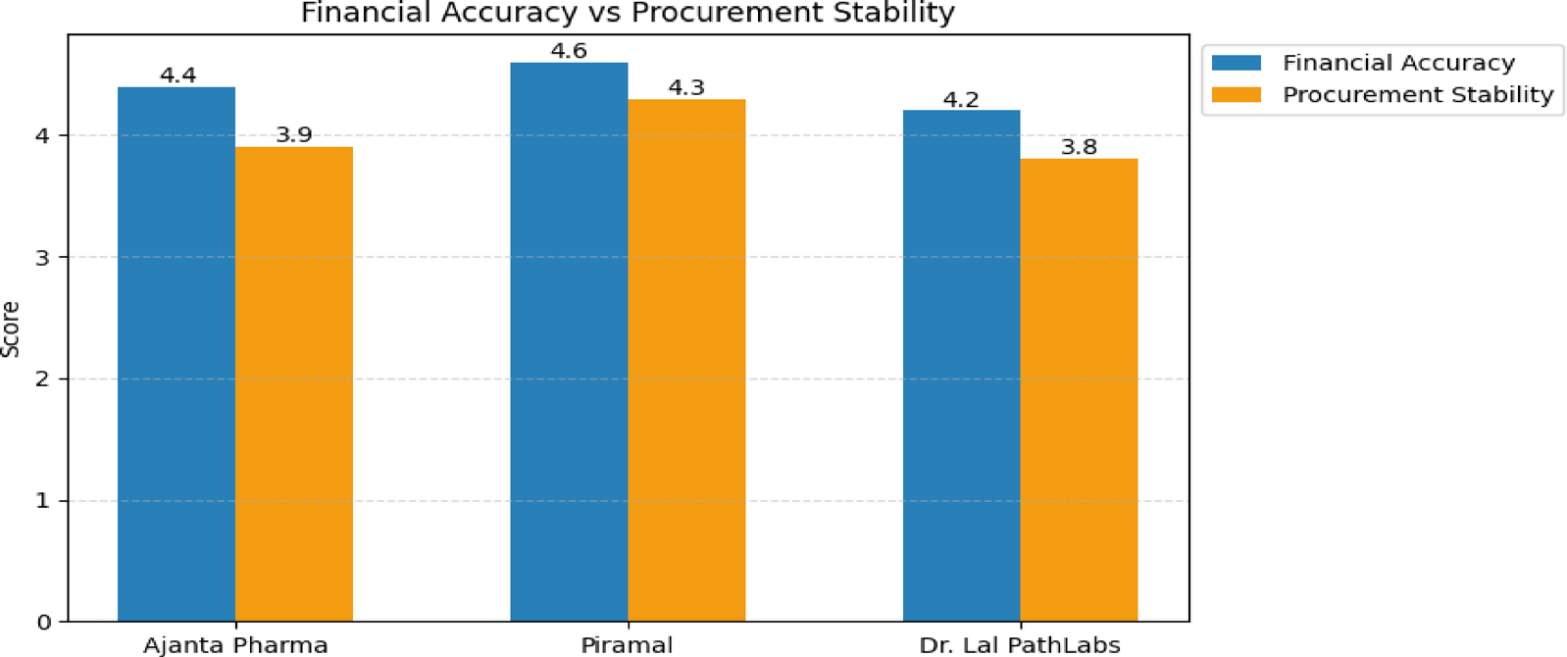
Financial Accuracy vs. Procurement Stability.

**Fig. 11:**
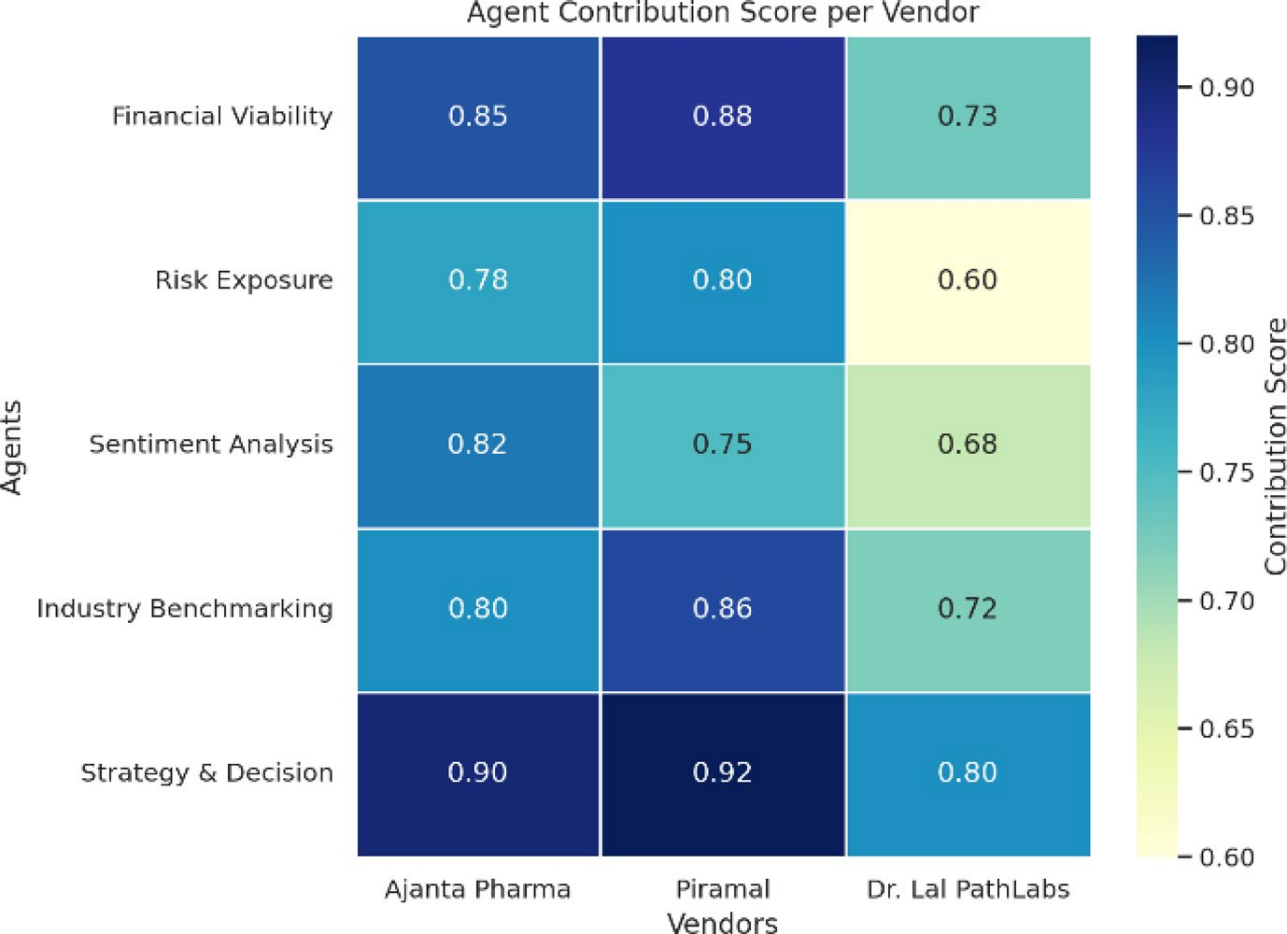
Heatmap of agent contribution scores per vendor (generated using Python 3.10 and Seaborn 0.13.2).

The bar chart presented in Fig. 10 indicates the relationship between the level of accuracy with which vendors register financial transactions and the level of the issues of the procurement with them. Piramal has enhanced financial accuracy (4.6) which ensures that there is more accuracy in the screening of suppliers compared to the uncertain purchasing by Dr. Lal PathLabs (3.8). Accurate visualization is ensured by data labels and chart legend remains separate out of the way to not confuse the reader. Figure 11**.** illustrating the heatmap of agent contribution scores per vendor across five agent types: Financial Viability, Risk Exposure, Sentiment Analysis, Industry Benchmarking and Strategy & Decision. The heatmap was generated using Python (version 3.10) with the Seaborn library (version 0.13.2), available at https://seaborn.pydata.org.

Piramal consistently receives high contribution scores across all agents, particularly in Strategy & Decision and Industry Benchmarking, indicating strong overall alignment with multi-agent assessments. Ajanta Pharma also performs well, especially in Strategy & Decision and Financial Viability, though slightly lower than Piramal in benchmarking and risk. Dr. Lal PathLabs, on the other hand, shows the lowest contribution scores across most agents, particularly in Risk Exposure, suggesting weaker performance and higher uncertainty. The visualization highlights Piramal as the most balanced and highly rated vendor across diverse evaluation criteria, supporting its selection in strategic procurement decisions.

### Empirical benchmarking & real-world validation

In addition to calculations, each vendor’s recommendation is verified with real-world purchasing simulations and compared with a company’s past experiences.

To judge vendor choices with AI, the framework looks at previous procurement cases, reviews a supplier’s finances, operations and levels of consistency in their sentiment.

1. Findings in research :

- What happened to Ajanta Pharma’s finances was accurately predicted, thanks to procurement liquidity remaining in line with past patterns.
- By selecting vendors with a low-risk deviation of 0.761, Piramal showed its recommendations were reliable financially and with regard to strategy.
- According to the analysis, Dr. Lal PathLabs had a sentiment score of 0.0048 that agreed with the market’s reputation trends and the suppliers’ assessments.

2. The use of case studies and procurement simulations is very helpful.

- Live playing of simulations improved how efficiently strategic sourcing works by suggesting vendors with AI.
- Ajanta Pharma is focused on making sure suppliers were dependable which helped to maintain wintergreen AI-based confidence.
- In scoring suppliers, Piramal valued how well they could pay and whether they would honor their promises.
- Dr. Lal PathLabs responded quickly to changes by using AI for insights, matching its sourcing with the ups and downs in the market.

### Model accuracy

They are used to objectively confirm the accuracy of the products chosen and how consistent the delivered information is. As follows.

1. BLEU Score ensures that AI-generated financial insights accurately and clearly recommend the right decisions for users. The equation is shown on (2)

2 $${\text{BLUE = BP}}{\mathrm{.exp}}\left( {\sum\limits_{n = 1}^{N} {\omega_{n} .\log p_{n} } } \right)$$

where: *N* = maximum n-gram order (e.g., 4 for BLEU-4).

$\omega_{n}$ = weight for each n-gram (e.g., $\omega_{n} = \frac{1}{N}$ ).

$p_{n}$ = precision of n-gram of order nnn.

BP = brevity penalty.

2. METEOR Score evaluates the linguistic suitability and technical correctness displayed throughout multi-agent recommendation outputs as shown in Eq. (3)

3 $${\mathrm{METEOR}}\; {\mathrm{Score}} = \frac{{{\mathrm{Matching}}\; {\mathrm{Words}}}}{{{\mathrm{Weighted}}\; {\mathrm{Average}}\; {\mathrm{Length}}}} \times {\mathrm{Penalty}}\; {\mathrm{Factor}}$$

where: Matching words = Exact or stemmed matches between reference and generated text.

Weighted average length = Adjusts for readability consistency.

Penalty factor = Punishes excessive fragmentation in generated text.

3. BERT Score: Evaluates whether stock selection recommendations maintain semantic continuity and accuracy as shown in Eq. (4)

4 $${\mathrm{BERT}}\,{\mathrm{Score}}\, = \frac{{\sum_{ij}^{N} max\,Cosine\,Sim\left( {reference_{i} ,generated_{j} } \right)}}{N}$$

where: Cosine Sim = Semantic similarity between reference and generated recommendation embedding’s.

*N* = Number of tokens in the generated recommendation.

4. Cosine Similarity: Establishes the correlation between the content produced and the content created by financial experts is explained in Eq. (5)

5 $${\text{Cosine }}\,{\mathrm{Similarity}} = \frac{A \cdot B}{{\left\| A \right\|\left\| B \right\|}}$$

where: A, B = Vectorized representations of reference and generated recommendations.

## Conclusion

This framework uses AI to help with comprehensive intelligence in purchasing, improve how vendors are assessed, evaluate risks and plan strategic sourcing. With help from organized financial information, sentiment analysis and competitor assessment, the system reduces the effort needed for vendor selection and supports purchase choices based on data. Nevertheless, there are areas where the framework can be improved, for example by relying on data, having added computation costs and being susceptible to sentiment approaches and hard to adjust in scenarios with difficult changes in the market. Since suppliers in the system rely on each other, it can lead to delays in decision-making, so continued oversight is needed to keep estimates precise and the process effective. To make MAS-based vendor selection more precise, stronger data validation steps, better processing speed and continual real-time updating are needed. Making sentiment interpretation stronger and including additional risk factors will increase the system’s skill in responding to supply chain challenges and new trends in procurement. Enhancements in the future might include using deep learning for supplier profiles, adaptive reinforcement learning for procurement choices and creating MAS systems using a combination of tools, keeping the framework dependable, flexible and able to work with fast-changing supplier environments.

In the point of limitations, the accuracy scores for this model in financial situations are impressive, issues remain. Structured finance data, sentiment analysis and comparison to competitors are main parts of the system, but they can be flawed because of missing information, old information and unreliable news. If data is incorrect or missing, it can direct procurement strategies toward wrong choices about vendors which may lead to altered assessments. Although MAS helps evaluate suppliers faster, using both parallel and hierarchical methods, needing other agents to act can sometimes clog the process. Delays or inaccurate information from the Financial Viability, Risk Exposure or Sentiment Agents may result in the Strategy & Decision Agent issuing recommendations that aren’t up to standard. Because huge volumes of financial data are processed, latency in making decisions can go up due to the high computing power needed. Llama 2 works faster when local, yet challenges with data performance may stop it from being used for fast comparisons of several metrics from various vendors. Although sentiment analysis helps judge vendors, feelings, possible biases and public dramatization may cause wrong judgments. If a vendor has a market incident or PR problem that is later forgotten, unjustified decisions for not selecting the vendor may still be made during purchasing.MAS handles regular financial information well, but events such as global issues, new regulations and supply difficulties can move more quickly than the system can keep pace. The framework depends on the system’s capability to learn new things, as risks may change rapidly in the industry. If the Multi-Agent System is not fine-tuned regularly, it might not be able to match the latest trends in procurement and suggest less accurate vendors.

## Data Availability

The datasets analysed during the current study are publicly available at 10.31219/osf.io/gdvbj_v1, https://arxiv.org/abs/2501.05468, and 10.1038/s41598-025-98483-1. No new datasets were generated or analyzed during this study.
